# Watch the Effect

**Published:** 1890-06

**Authors:** Edward Cranch

**Affiliations:** Erie, Pa.


### WATCH THE EFFECT.

Edward Cranch, M. D., Erie, Pa.

Watch the effect! The whole sermon is in that little phrase, now see how it unravels. Who will admit that he does not watch the effect of his remedies, and who will not say, with more or less pride (perhaps, as I have heard it, with applause from students), "I cure my patients"? Yet the same professor, years after, said in my hearing, "now, *since I have kept a record of my cases,* I find the practice of medicine very different, and much more satisfactory than ever before." No one is very ready to admit that most of his patients "get well" without his direct assistance—that is, that the medicines had little or nothing to do with their recovery—yet this is the case in a surprisingly large number of prescriptions. Yet decided, even brilliant cures are not impossible, and nothing is more pleasant in a physician's experience than to watch the effect day by day of a well-chosen, unchanged-from, and unrepeated remedy, as the vital force it has roused gradually but surely throws off disease, with all its lugubrious and discomforting accompaniments.

One who is accustomed to observe effects, will not give medicines and change them the same day merely because he cannot see instant improvement on the lines that he or his books have laid down for that remedy to act upon; he will question every phase of the case, and if the answer is, no worse anywhere, or if worse, only in the line of the pathogenetic effect of the given drug, then the observer will wait, with plenty of resolution, plenty of alertness, and above all, plenty of "Sac-lac.": not to deceive the patient, but to keep off discouragement and untimely argument. Every one knows that physicians give placebos, but no one likes to think that he or she is taking them, and when one is sick one is in no mood to argue the question.

So it is well to have several vials of placebo scattered through your case, besides a separate case containing *only* placebos all carefully labeled, and composed only of Sac-lac., or alcohol—not like the so-called blank pills of Dr. Cathell, in *The Physician Himself,* who in Chapter V of a most excellent work (but one very hard on the homœopathic frauds, while sufficiently courteous to the honest homœopathicians) advocates the use of pellets soaked in *fluid extract of belladonna,* or in *compound tincture of iodine,* as placebos!

One accustomed to watch his patients more than his books, will be slow to report cures till he is sure of them, for often some circumstance or condition, unknown or unexplained at the time, shows that, after all, the medicine given had little, if anything, to do with the cure, or else the supposed cure was not a cure at all, but only a remission, or a piece of concealment, or of insufficient investigation.

If a certain learned professor had asked more closely after one of his patients, he would not have told his class that Arsenic was good for a "very peculiar neuralgia of the tongue, like red-hot needles all through one side of it, coming in agonizing paroxysms," going on to say that he knew of a case recently helped thereby.

Of course, I believed him, and made a note of it, but soon after, on coming to Erie, I was introduced to this very case by the physician in charge, who said that Dr. ——, naming the professor, had been consulted, and recommended Arsenic, which he was confident would help, but it *did not*, and, what is worse, nothing did, for the patient went on worse and worse up to her death, which occurred some three or four years later. No post-mortem was held, but it was a most inveterate case of tic douloureux, probably from some tumor in the brain or orbit. Often I have begun to think my prescription was doing wonders, only to find, later, that the patient had either not taken it at all, or had been using other remedies at the same time, making the whole case uncertain. Or a sore throat would look diphtheritic and be well with most surprising promptness, which would be duly credited to the remedy, till a succession of such cases showed plainly the existence of an epidemic tonsillitis, with exudation, but not true diphtheria, and many cases got well just as promptly without medicine. The late epidemic, "La Grippe," yielded many such experiences, so that it was hard to tell just what the remedies *did* do.

But the most uncertainty comes from not waiting long enough on one remedy, and changing or repeating on insufficient indications, especially in acute cases, where much valuable time is often lost in the endeavor to gain time, forgetting that the medicine must *always* have sufficient time to act, and that "no worse," or "a little better," if substantiated on careful interrogation of every known vital indication, is the signal for placebo, not to be changed heedlessly; then, at least, if the case is not made, it is not marred, and it has the best known chance of getting well.

When any medicine is indicated, it is generally plainly so; if we have to imagine that symptoms are present, the chances are that no drug at all is needed at first; if it is, the fact will soon become apparent, and the indications clear. Then, if undue haste or carelessness does not spoil the progress, the result is sure. One case of typhoid fever will illustrate this: W. K. T., age twenty-four, tobacconist, complained merely of weakness and loss of weight, for which a careful diet was directed; three days later he developed typhoid fever, with symptoms calling for Bryonia. This was given in Tafel's 1,000th, in water, for three days, when a slight but decided amelioration occurred, and placebo was substituted. The tongue continued dry, and the bowels constipated, for two weeks, but no more medicine was given, and the patient progressed to full recovery in the most satisfactory manner, without another dose of any medicine, except one dose of Sulphur, given for occasional faintness, with burning of the feet, in the fourth week when he was beginning to walk about the room.

Several other cases in the neighborhood, treated by antipyrin and chloral, died with cyanosis, or came so near death that many gave them up and then when medicine was suspended they recovered! In all my cases this winter I saw not one where pneumonia supervened after grip or anything else, and I lost no case of typhoid fever or of grip.

A case of mania will do to close with: A lady imagined herself mesmerized by her absent pastor, and was in a great fright. Aconite$^{1000}$ quieted her, but did not remove the delusion, which soon mingled with others—that every one was possessed of the devil, that her eyes were strangely wild, and that nothing was as it should be. Her face was very red, but there was no headache or other ascertainable symptom. Melilotus$^{30}$ was given in water, for two days, with most happy effect, improvement was rapid, the illusions faded, and a state of delightfully restful sleepiness came on, when the medicine was discontinued, till, in less than a week more, she voluntarily quitted the bed, saying she was now quite rested, and did not believe that her fancies, which she was fully aware of, would return, nor have they at present writing.

